# Reconstruction of Scapula Bone Shapes from Digitized Skin Landmarks Using Statistical Shape Modeling and Multiple Linear Regression

**DOI:** 10.1007/s10439-025-03768-1

**Published:** 2025-06-19

**Authors:** Augusto Marques, João Folgado, Carlos Quental

**Affiliations:** IDMEC, Instituto Superior Técnico, Universidade de Lisboa, Av. Rovisco Pais, 1, 1049-001 Lisbon, Portugal

**Keywords:** Scapula, Statistical shape model, Computational modeling, Subject-specific, 3D shape reconstruction, Bone landmark prediction

## Abstract

**Purpose:**

The aim of this study was to develop an algorithm for the reconstruction of scapula bone shapes from skin landmarks, using a statistical shape model (SSM).

**Methods:**

A sample of 56 scapula segmentations was used, as well as 4 scapular bone and skin landmarks. Regression models were built to predict the coordinates of bone landmarks from skin landmarks using subject-specific variables, namely skin landmark coordinates, sex, age, weight, and height. The scapula shapes were reconstructed by fitting the bone landmarks of the SSM’s mean shape to the predicted bone landmarks of the subject.

**Results:**

The developed regression models registered a *R*^2^ ranging from 0.70 to 0.98, with a maximum median error of 4 mm. The average surface-to-surface errors were equal to 2.41 and 2.45 mm using digitized and predicted bone landmarks, respectively. No significant statistical differences were observed between scapula shapes reconstructed from digitized and predicted bone landmarks.

**Conclusion:**

This study demonstrated the reliability of the developed algorithm in deriving subject-specific scapula shapes from experimentally acquired data, highlighting that scapula shape reconstructions based on a limited set of landmarks can effectively generate subject-specific computational models without the need for additional medical imaging.

**Supplementary Information:**

The online version contains supplementary material available at 10.1007/s10439-025-03768-1.

## Introduction

The development of high-fidelity computational biomechanical models involves several time-consuming and labor-intensive steps, the most critical of which including segmentation; geometry reconstruction; and geometry processing [4]. Statistical shape models (SSMs) enable the manipulation of biological shapes *in silico*, offering a valuable approach for developing subject-specific computational models [5]. In particular, they can act as a shortcut for generating subject-specific bone shapes by eliminating the need for steps like medical image acquisition and segmentation. These shapes are essential for accurate biomechanical analyses, such as in musculoskeletal (MSK) modeling and finite element analysis [2, 11, 26]. However, a comprehensive approach for the accurate, automated, and efficient generation of these biomechanical models remains missing, especially for the shoulder joint, which has been less studied compared to other regions of the human body [11, 24]. The reconstruction of subject-specific bone shapes from experimentally acquired data, typically comprising the positions of anatomical landmarks obtained through motion capture systems [18, 31], represents an important contribution to the field and a promising first step toward this challenge. Nevertheless, since the positions of anatomical landmarks are typically collected from markers placed on the skin, they may not fully represent the underlying bone structures. Hence, transforming these skin landmarks into bone landmarks, which can be used for shape reconstruction, is of utmost importance [12, 18].

To date, the reconstruction of scapula shapes from skin landmarks using SSMs has not yet been explored. As with other well-studied anatomical structures, such as the lumbar spine, pelvis, femur, and tibia [9, 19], SSM-based shape reconstructions of the scapula relied exclusively on bone landmarks extracted from medical images. Salhi et al. [23] and Huang et al. [10] reconstructed scapula shapes from predefined sets of bone landmarks, demonstrating the influence of landmark location on reconstruction accuracy. However, these studies used virtual landmarks, meaning they did not account for whether these points could be reliably identified in laboratory settings or consistently reproduced in medical imaging data, limiting their applicability to real-world scenarios. Mutsvangwa et al. [17] improved upon this by using reproducible X-ray-derived bone landmarks, but the challenge of reconstructing scapula shapes from skin landmarks, such as those obtained from motion capture systems, remains unresolved.

Extending SSM-based reconstruction to skin landmarks introduces additional complexity due to soft tissue artifacts, which have been a key challenge in musculoskeletal modeling. Few studies have addressed this issue and those that have focused exclusively on the lower limbs. Nolte et al. [18] and Asvadi et al. [1] investigated the feasibility of reconstructing femur shapes from skin landmarks, considering different approaches to account for soft tissue artifacts. Nolte et al. [18] developed linear regression models to estimate soft tissue thickness between skin and bone, whereas Asvadi et al. [1] evaluated reconstructions based on either an average soft tissue thickness or subject-specific measurements from CT data. Their findings were mixed: Nolte et al. [18] reported that skin-based reconstructions were comparable to those derived from bone landmarks, while Asvadi et al. [1] observed significant errors, particularly when landmark positioning was inaccurate. These studies highlight the need for careful skin landmark selection and strategies to mitigate soft tissue artifacts. For the scapula, whether accurate reconstructions can be achieved from skin landmarks remains an open question.

While no previous studies have explored scapula reconstruction from skin landmarks, addressing this challenge could significantly enhance subject-specific modeling for biomechanical applications. The aim of this study was to develop and validate an algorithm for the reconstruction of scapula bone shapes using coordinates from a limited set of digitized skin landmarks, typically acquired via motion capture systems, and a SSM of the scapula. This research represents an important advancement in scapula reconstruction using SSMs, achieving two key milestones:

1. Introducing a novel method, hereafter referred to as “skin-to-bone regression model,” for transforming scapular skin landmark coordinates into scapular bone landmark coordinates. This model, developed through stepwise multiple linear regression, builds upon previous works by Meskers et al. [15], Lalonde et al. [12], and Nolte et al. [18];
2. Integrating a SSM of the scapula with the skin-to-bone regression models into a reconstruction algorithm capable of generating a subject’s right scapula shape from experimentally acquired subject-specific data.

## Materials and Methods

**Fig. 1:**
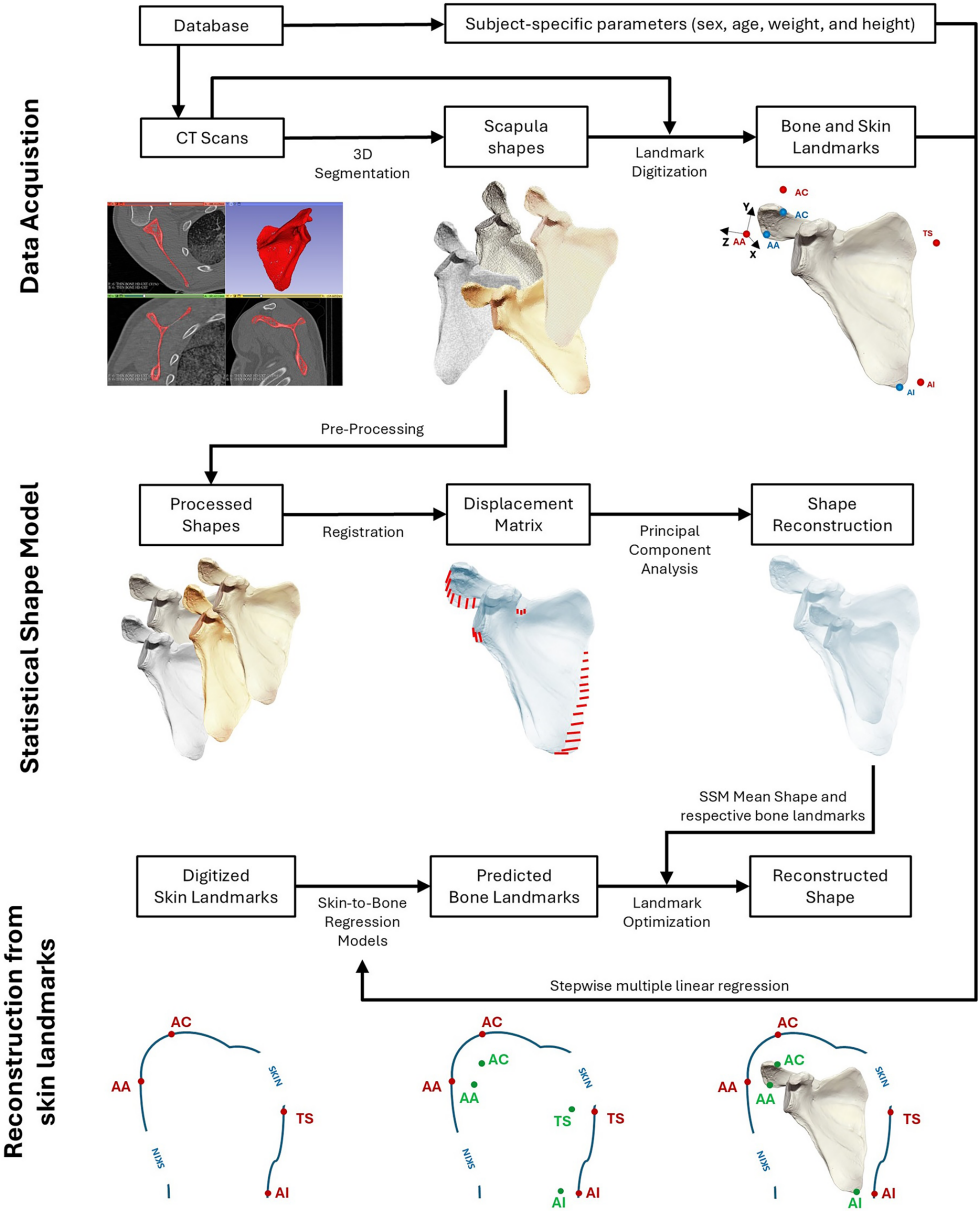
Overview of the methodology implemented in this study. The first step was the segmentation of scapula shapes and acquisition of subject-specific parameters, namely sex, age, weight, and height. Bone and skin landmarks were acquired using both the shapes and CT scans. Scapula shapes were processed and registered, allowing shape reconstruction via PCA. The coefficients of the skin-to-bone regression models were computed from subject-specific parameters and bone/skin landmarks using a stepwise model selection process. Reconstruction from skin landmarks was implemented via landmark fitting between the SSM mean shape’s bone landmarks and the subject-specific predicted bone landmarks

The method implemented in this study, summarized in Fig. 1, combined mathematical algorithms such as principal component analysis (PCA) and linear regression to compute subject-specific scapula shapes solely from 3D coordinates of skin landmarks and subject characteristics, such as sex, age, weight, and height. To achieve this, the necessary data, including scapula segmentations and corresponding bone and skin landmarks, were acquired and used to develop a SSM of the scapula and regression models mapping skin landmarks into bone landmarks. These models were integrated in an algorithm that computes the best reconstructed shape by fitting the bone landmarks of the SSM’s mean shape to a subject’s bone landmarks, predicted by the skin-to-bone regression models. The algorithm was validated by comparing predicted bone landmarks with digitized bone landmarks (manually marked in the CT scans) and the reconstructed shapes with ground truth shapes (resulting from the segmentation procedure).

### Dataset Description

The dataset of DICOM imaging data used was obtained from the New Mexico Decedent Image Database (NMDID) [6]. Each subject is represented by approximately 10.000 images, with a slice thickness of 1 mm with 0.5 mm overlap and an in-plane resolution of 0.88 mm by 0.88 mm. From these, only the CT scans of the upper body were used, between the abdomen and head. Subjects with CT scans showing metallic implants, bone fractures, bone defects, or artifacts that lowered the segmentation quality were discarded. Subjects were chosen randomly from three samples, each composed of approximately 160 decedents, associated with three age ranges (20–40, 40–60, and 60–80). A total of 56 subjects were included in this study. Data regarding each subject were also obtained, namely their sex, age, weight, and height. Table 1 shows statistic measures regarding these parameters.

**Table 1:** Frequency, mean, and standard deviation of the sex, weight, height, and age distributions for the sample of subjects considered

Age range	# Subjects	Male	Female	Weight (kg)	Height (m)	Age (years)
20-40	17	7	10	80.3 ± 16.0	1.72 ± 0.08	28 ± 5
40-60	18	8	10	75.4 ± 16.6	1.72 ± 0.08	49 ± 7
60-80	21	8	13	71.0 ± 14.8	1.70 ± 0.07	68 ± 5
All	56	23	33	75.3 ± 15.9	1.71 ± 0.08	50 ± 18

### Data Processing

To obtain 3D shapes from the DICOM imaging data, segmentation was performed in 3D Slicer [7]. Only right scapulae were segmented. Bone and skin anatomical landmarks of the available segmentations were digitally acquired from both the segmented models and the corresponding CT data. In this study, “skin landmarks” refer to bone landmarks measured at the skin level, as commonly done in motion capture laboratories. Four landmarks were selected for the scapula, considering the recommendations from the International Society of Biomechanics (ISB) for the experimental measurement of upper limb motions [30]. These landmarks were the acromial angle (AA), the root of the spine (TS), the inferior angle (AI), and the acromioclavicular joint (AC). The coracoid process (PC) was excluded from the study because it is often challenging to palpate, especially in individuals with high body fat. To acquire coordinates of bone and skin scapular landmarks, the software nmsBuilder was used [27]. The acquisition was made individually for each of the 56 scapula shapes segmented. The landmarks’ coordinates were transformed into the body-fixed reference frame of each scapula, defined according to ISB recommendations using skin landmarks [30]. This transformation allowed a consistent description of coordinates among the different scapula shapes. The population of shapes and landmarks was divided in a 70–30% ratio, such that 39 samples (70%) were used to develop the SSM and the skin-to-bone regression models, and 17 samples (30%) were used to assess the performance of the reconstruction algorithm developed.

### Statistical Shape Model

For computational simplicity, the 3D scapula shapes were decimated and remeshed to have 6000 isotropic vertices. The SSM of the scapula was developed in three stages: first, a rigid registration of the shapes with a randomly selected reference shape; second, a non-rigid registration to achieve point-to-point correspondence; and third, an extraction of the mean shape and shape variations of the scapulae through PCA. Standard performance metrics described in the literature, such as generalization, specificity, and compactness, were considered for the validation of the SSM [9]. The Supplementary Material provides detailed information on all stages of the development of the scapula SSM.

### Skin-to-Bone Regression Models

#### Data Analysis

The dataset used to build the skin-to-bone regression models comprised 39 samples of two sets of variables—12 response variables and 17 predictors. The response variables were the *x*, *y*, and *z* coordinates, in the scapula’s body-fixed reference frame, of the four bone landmarks considered. This resulted in a total of 12 independent skin-to-bone regression models. The predictors were the *x*, *y*, and *z* coordinates, in the scapula’s body-fixed reference frame, of the four skin landmarks considered, the distance between each pair of skin landmarks, and the subject data previously acquired (sex, age, weight, and height). Body mass index (BMI) was also computed since it represents a non-linear relationship between weight and height. Since the transformation of coordinates to the body-fixed reference frame of the scapula resulted in some skin coordinates being equal to zero, they were discarded as predictors. These were the *x* coordinates of AA, TS, and AI; the *y* coordinates of AA and TS; and the *z* coordinate of AA.

#### Model Selection

To decrease the probability of overfitting or underfitting scenarios, the dataset was initially divided into 10 random subdatasets (or folds), using a shuffle split method. Each subdataset was subjected to a train–test–split with a 70–30% ratio. The shuffle and train–test splits were implemented using the *scikit-learn* Python library [20]. This way, each regression model, for a given response variable, was computed ten times. Skin-to-bone regression models, one for each bone landmark coordinate, were constructed via a stepwise selection method with an entry and removal *F*-test *p*-value of 0.05 and 0.10, respectively [13]. The stepwise multiple skin-to-bone regression models were obtained using the SPSS software (IBM Corp., NY, USA). The best regression model, over the 10 possible models for landmark *i* and respective coordinate *j*, was chosen according to an evaluation score, calculated for each fold, based on its training and testing mean absolute errors (MAEs) given by

1 $${\mathrm{MAE}}_{ij}^{\mathrm{train}}=\frac{1}{27}\sum_{k=1}^{27}\left|{y}_{ijk}^{\mathrm{train}}-{\widehat{y}}_{ijk}^{\mathrm{train}}\right|,$$

2 $${\mathrm{MAE}}_{ij}^{\mathrm{test}}=\frac{1}{12}\sum_{k=1}^{27}\left|{y}_{ijk}^{\mathrm{test}}-{\widehat{y}}_{ijk}^{\mathrm{test}}\right|,$$

where ${y}_{ijk}$ and ${\widehat{y}}_{ijk}$ are the real and predicted *k*th instances for landmark *i* and coordinate *j*, respectively. The evaluation score of a regression model, ${s}_{ij}$, was computed as

3 $${s}_{ij}=\frac{1}{2}\left[\frac{{\mathrm{MAE}}_{ij}^{\mathrm{train}}+{\mathrm{MAE}}_{ij}^{\mathrm{test}}}{2}+\left|{\mathrm{MAE}}_{ij}^{\mathrm{train}}-{\mathrm{MAE}}_{ij}^{\mathrm{test}}\right|\right].$$

The first term of the evaluation criterion accounts for the average magnitude between the training and testing MAEs, whereas the second term penalizes the absolute difference between the two, to avoid under and overfitting scenarios. For each landmark *i* and respective coordinate *j*, the regression model with the lowest ${s}_{ij}$ was selected as best, among the 10 models developed. Knowing that *i* ∈ {AA, TS, AI, AC} and *j* ∈ {*x*, *y*, *z*}, Eq. (3) was applied to all possible combinations (*i*, *j*), and the 12 best skin-to-bone regression models were obtained.

### Scapula Reconstruction from Landmarks

#### Conceptualization

Using the information from both the SSM and the skin-to-bone regression models, an algorithm was developed in Python to reconstruct a subject’s scapula shape using only the coordinates of four skin landmarks and subject characteristics such as sex, age, weight, and height.

Initially, the algorithm takes in the user’s input data and proceeds to transform the skin coordinates into the body-fixed reference frame of the scapula, defined according to ISB [30]. These body-fixed skin landmark coordinates, along with the subject’s data, are then utilized to predict the coordinates of bone landmarks. The predicted bone landmark coordinates are computed using the best estimated regression coefficients, for each landmark and coordinate, obtained via the procedure outlined in "Model Selection" section. The predicted bone landmarks serve as the SSM’s reconstruction target, henceforth designated as *target*.

To identify the best reconstructed shape that fits the target, an optimization procedure was adopted. By minimizing a distance metric criterion between the reconstructed and target shapes, given by

4 $$f\left({\boldsymbol{\upkappa}}\right)={\Vert {\mathbf{x}}_{\mathrm{target}}-\mathbf{x}\left({\boldsymbol{\upkappa}}\right)\Vert }^{2}+\sum_{m=1}^{M}\left[{\mathrm{e}}^{{\kappa }_{m}^{2}}-1\right],$$

the optimal vector ${{\boldsymbol{\upkappa}}}^{*}$ is sought. The components ${\kappa }_{m}$ represent the weights in the linear combination of the first *M* modes of variation that defines the SSM shape (see the Supplementary Material for further detail). In Eq. (4), the first term emphasizes the alignment between the bone landmarks of the reconstructed shape and the target, while the second term penalizes the decision variables, which are normally distributed. Solutions with higher ${\kappa }_{m}$ are less likely to be optimal, since the shape variation introduced by these is less likely to belong to the population of ground truth scapula shapes. The − 1 term ensures that the fitness function remains the same when ${\kappa }_{m}=0$, since it does not introduce variation into the shape. For the calculation of the distance metric in Eq. (4), the iterative closest point algorithm was applied to ensure a proper alignment between the reconstructed and target shapes. The optimization problem was solved through the application of a genetic algorithm (GA) followed by a truncated Newton constrained (TNC) algorithm to assure the solution found was, at least, a local minimum. The GA and TNC algorithms used were implemented in Python via the *pygad* [8] and the *scipy* [29] libraries, respectively.

#### Performance Evaluation

Scapula shapes were reconstructed from digitized and predicted bone landmarks, for each of the 56 available shapes, to evaluate the performance of the reconstruction algorithm. For each scenario, two metrics were computed based on previous literature: the landmark-to-landmark (L2L) and the surface-to-surface (S2S) errors [3, 16, 17, 19]. The L2L error is the RMSE, in mm, between a bone landmark of the optimal reconstructed shape and the corresponding bone landmark of the target. This measure is useful for the evaluation of the GA and TNC algorithms, since it is related to the objective function, defined in Eq. (4). The S2S error represents the point-to-point RMSE between the optimal reconstructed shape and the ground truth shape. It was calculated using point-to-point correspondences across all vertices of the scapular surface, serving as a measure of the overall geometric similarity between the two shapes. Hypothesis tests were performed, for L2L and S2S error distributions, to further analyze the statistical significance of the difference between the results computed with digitized and predicted bone landmarks. The goal was to infer on the meaningfulness of the skin-to-bone regression models in the reconstruction algorithm. The non-parametric Kruskal–Wallis test was implemented at a 5% significance level. The null hypothesis states that no difference exists between the L2L or S2S error distributions across groups, i.e., across digitized and predicted bone landmarks. *p*-values less than 0.05 reject this hypothesis, concluding that the L2L or S2S errors distributions are statistically different. The Kruskal–Wallis tests were performed using the SPSS software.

## Results

### Statistical Shape Model

#### Shape Variation

**Fig. 2:**
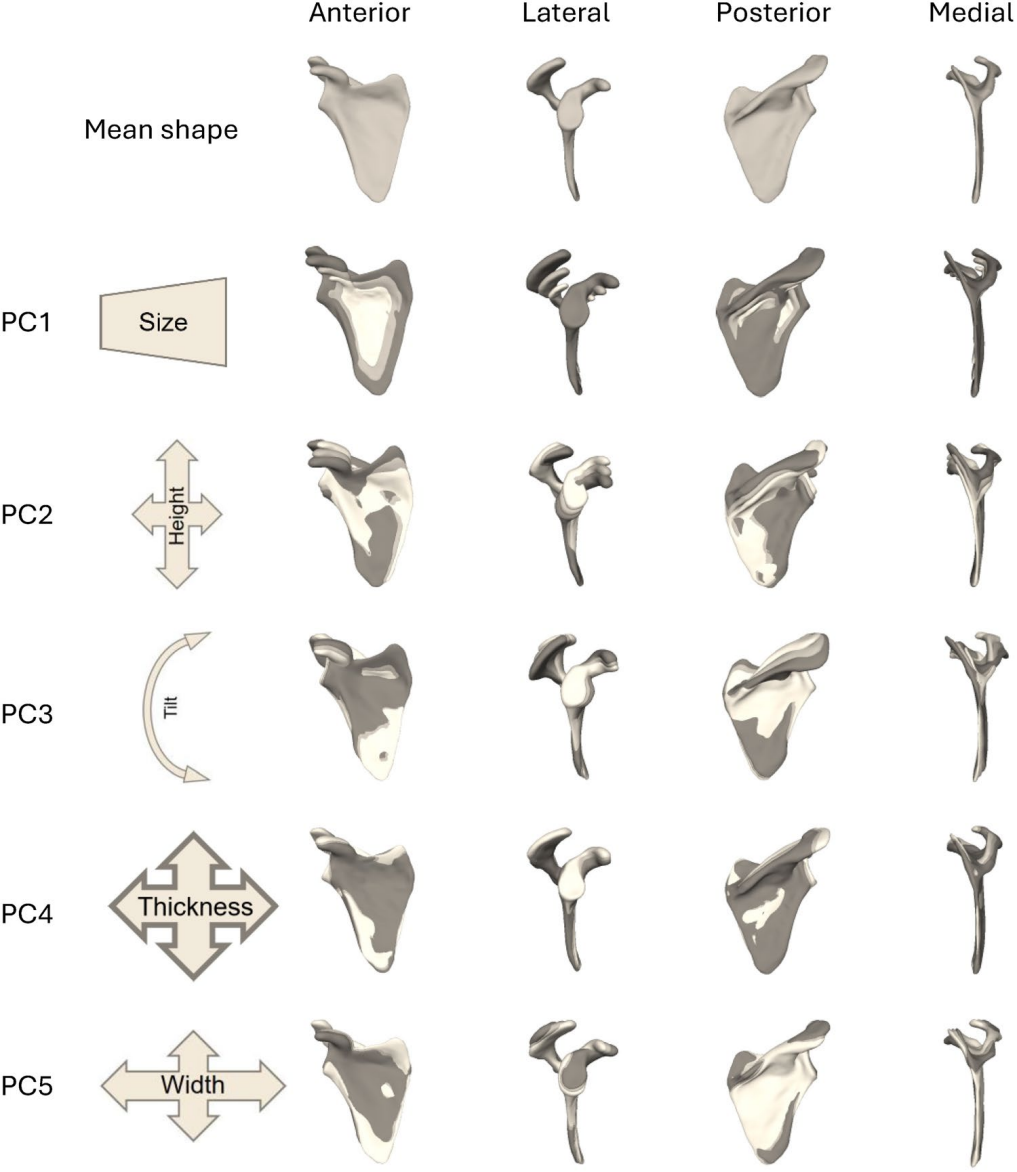
Pattern of scapula shape variation, introduced by the SSM, for the first five principal components. Each principal component is shown in four views, with the variation shown as three superimposed shapes—mean shape (light gray) as well as mean shape with − 3 (white) and + 3 (dark gray) standard deviation magnitudes

The mean shape of the SSM and respective modes of variation are depicted in Fig. 2. Five PCs were selected to show the SSM scapular variation since it was the number of PCs chosen for the reconstruction algorithm, based on the generality metric of the SSM. For brevity, the validation of the SSM is shown in Fig. S1 and discussed in detail in the Supplementary Material.

The first PC represents the uniform scaling of the scapula. The second PC is represented mostly by the height of the scapula. Note the increase in height, from − 3*σ* to + 3*σ*, in the superiormost point of the superior angle, located in the superior border. As this change occurs, the decrease in height of the glenoid complex, acromion, and the coracoid process also occurs, with the last one increasing in volume. The third PC depicts the tilting of the scapula about a medial–lateral axis, with higher inclination in the spine and coracoid process. The fourth PC represents the overall thickness of the scapula. This feature can be identified in anterior and posterior views, by the superimposition of the subscapular and infraspinous fossae of the dark gray scapula shape. In a posterior view, the thickness of the scapular spine’s root increases, the higher the standard deviation applied. The fifth PC depicts the change in width of the scapula spines, and consequent elongation of the acromion. A higher variation elongates the scapula spine, reducing its thickness. Consequently, the acromion becomes less curved, flattened, and shorter.

### Skin-to-Bone Regression Models

#### Regression Coefficients

Table 2 presents the 12 best stepwise multiple linear regression models, computed with the skin and bone landmark coordinates of 39 subjects. The coefficients of the predictors deemed significant in the stepwise selection process are shown with a precision of 3 decimal places for better visualization. The quality measures considered for the skin-to-bone regression models registered minimum and maximum ${R}^{2}$ of 0.70 and 0.98, respectively, and a maximum difference between ${R}^{2}$ and ${R}_{\mathrm{adj}}^{2}$ of 0.04.

**Table 2:**
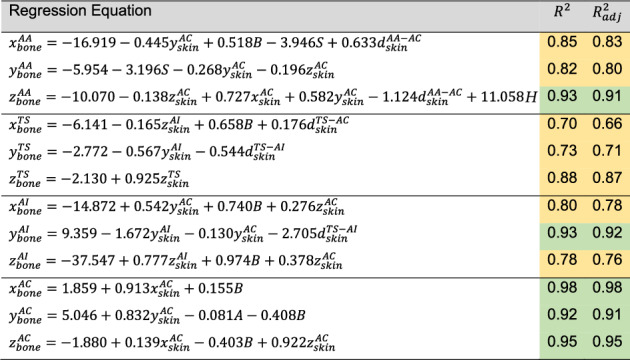
Coefficients and quality measures of the best skin-to-bone regression models

The symbols *A*, *B*, *H*, *S*, and *W* represent the age, BMI, height, sex, and weight variables, respectively. Female and male sexes are represented by 0 and 1, respectively. Quality measures lower and higher than 0.90 are represented in yellow and green, respectively

#### Bone Landmark Predictions

**Fig. 3:**
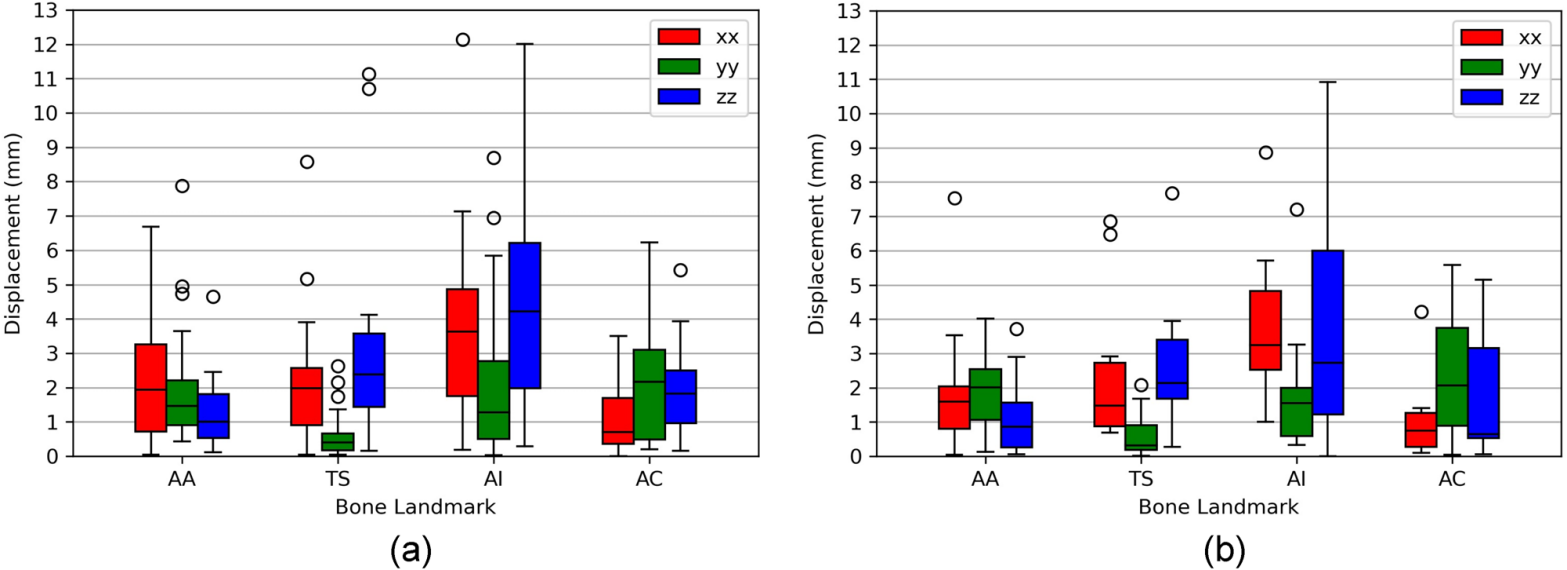
Prediction error distributions of the 12 best skin-to-bone regression models for the **a** training and **b** testing data. The error is represented as a displacement, in mm, between the predicted and digitized bone landmark coordinates

The skin-to-bone regression models, described in Table 2, were tested for the respective training and testing datasets. The models of the AA and TS landmarks showed median errors, in all three axes, below 2 and 3 mm, both for training and testing datasets, respectively (Fig. 3). The AC models presented similar performances, not surpassing a median error of 2.5 mm. The AI models presented the highest median error, for all three axes, both in training and testing datasets, reaching a maximum of approximately 4 mm. Most models presented outliers for either training or testing data, although in a low quantity.

### Scapula Reconstructions from Landmarks

#### Landmark-to-Landmark (L2L) Error

**Fig. 4:**
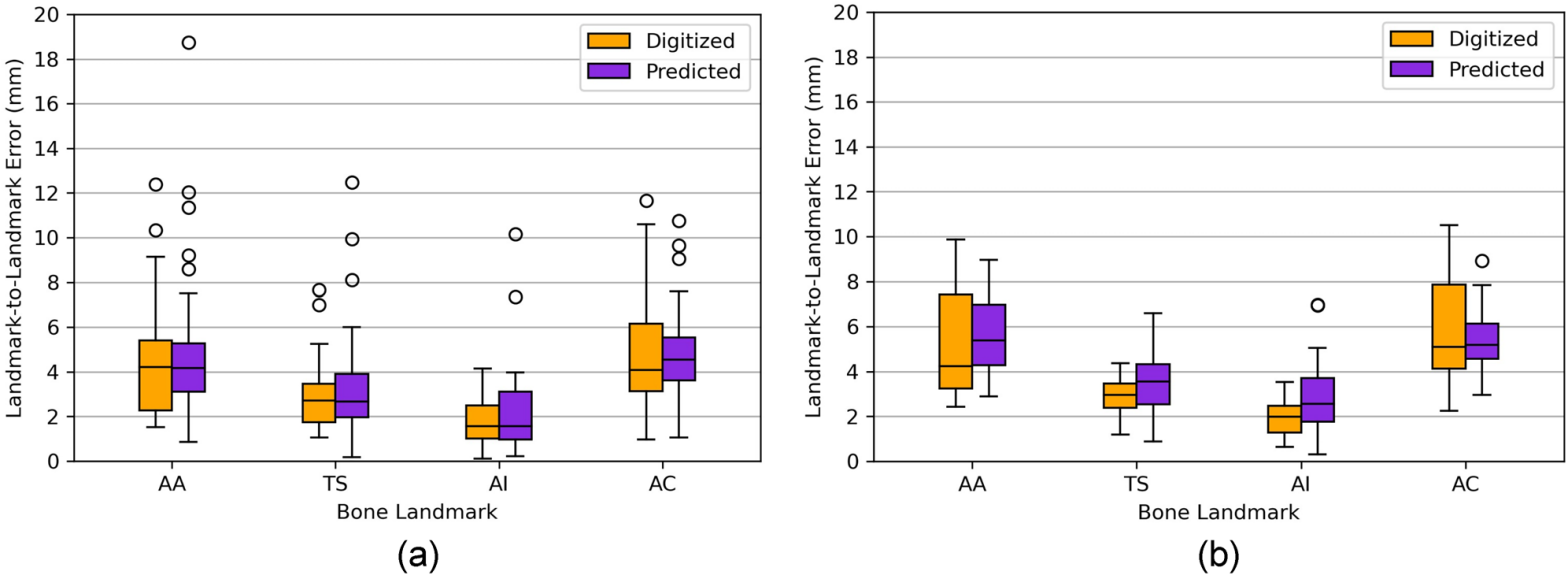
Boxplot distributions of the L2L error metric, resulting from the reconstructions built with digitized (orange) and predicted (purple) bone landmarks for the **a** train and **b** test datasets

The distributions of the L2L errors are depicted in Fig. 4. Few outliers are present, and median values are within 1 and 5 mm. A distinction in L2L error between the four landmarks can be identified for the AA and AC landmarks, which presented higher interquartile range (IQR) and respective whiskers.

The Kruskal–Wallis test employed showed no significant differences between the L2L error distributions obtained with digitized and predicted bone landmarks. *p*-values are provided in Table 3.

**Table 3:**
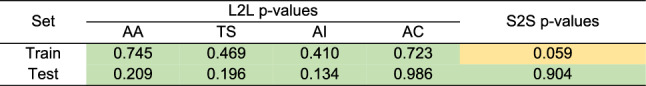
*p*-values of the Kruskal–Wallis test for the distributions of L2L and S2S errors, between digitized and predicted bone landmarks

Distributions are divided into training (39 subjects) and testing (17 subjects) sets. *p*-values higher than and closer to 0.05 are represented in green and yellow, respectively. *p*-values higher than 0.05 suggest the distributions of L2L or S2S errors obtained with digitized and predicted bone landmarks are not statistically different

#### Surface-to-Surface (S2S) Error

**Fig. 5:**
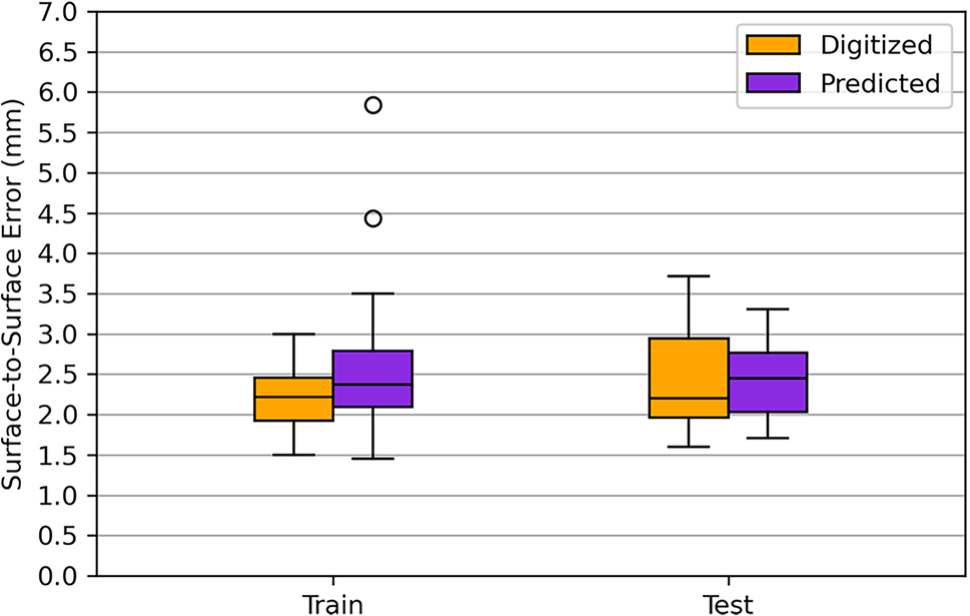
Boxplot distributions of the S2S error metric, resulting from the reconstructions built with digitized (orange) and predicted (purple) bone landmarks for the train and test sets

The S2S errors, which considers all the 6000 vertices that constitute the scapula shapes, and not only the 4 landmarks that were used to reconstruct them, were in a range between 1.5 and 3.75 mm (Fig. 5). The errors between training and testing datasets were within the same ranges, although those from predicted bone landmarks showed two outliers.

Like for the L2L errors, the Kruskal–Wallis test showed no significant differences between the S2S error distributions obtained with digitized and predicted bone landmarks (Table 3).

#### Best and Worst Reconstructions

**Fig. 6:**
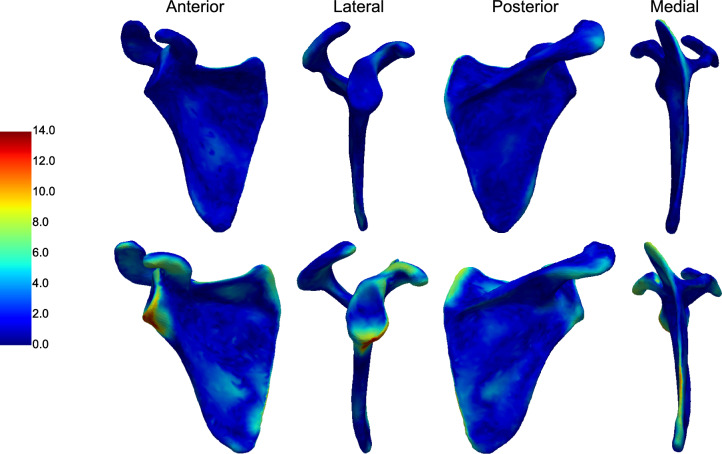
Heatmap of point-to-point distances, in mm, between the reconstructed and ground truth shapes for the best (top) and worst (bottom) scapula reconstructions, obtained with 5 PCs and from predicted bone landmarks

Figure 6 depicts heatmaps illustrating the point-to-point distances between the reconstructed and ground truth shapes for the best and worst cases. These cases correspond to the lowest and highest S2S errors registered in the test dataset, respectively, based on reconstructions from predicted bone landmarks. Figures with superimposed shapes for both scenarios are provided in the Supplementary Material (Fig. S2).

The reconstruction obtained for both subjects showed uniform and homogeneous forms, with the overall scapula shape preserved. For the best reconstruction, a higher degree of similarity was seen along the subscapular and infraspinous fossae, with point-to-point distances lower than 2.0 mm. Larger deviations were identified in the superior-anterior region of the glenoid cavity, reaching the superior face of the coracoid process. While the acromion did not perfectly match the ground truth shape, it maintained low point-to-point distances overall. The largest deviation in this region was centered at the AA landmark, with a distance of approximately 5 mm. The superior angle region showed a descending gradient along the medial border, with a maximum point-to-point distance of 8.1 mm at the superiormost point.

For the worst reconstruction, the distribution of higher point-to-point distances, depicted in green and yellow, was more prominent. Both the superior and inferior faces of the coracoid process, the superior region of the medial border, and the region around the glenoid neck showed deviations between approximately 5.0 and 9.0 mm. The largest discrepancy, peaking at 13.4 mm, was located in the antero-inferior border of the glenoid cavity.

## Discussion

Using statistical shape modeling, this study presented an innovative reconstruction algorithm capable of reconstructing scapula shapes from a limited set of skin landmarks and subject characteristics, including sex, age, weight, and height. A key contribution is the development of regression equations that transform skin landmarks into bone landmarks on the scapula, using variables derived from experimentally acquired subject-specific data. The reconstruction algorithm demonstrated good accuracy, as evidenced by low landmark-to-landmark (L2L) and surface-to-surface (S2S) errors across both training and testing datasets.

The number of PCs to retain in SSMs is typically determined based on a predefined percentage of cumulative variance [9]. However, this approach is highly dependent on sample size [14]—for a fixed cumulative variance threshold, the number of PCs to retain increases with sample size, potentially introducing noise from PCs that do not represent meaningful anatomical variation. In this study, although 95% of the variability in the developed scapula SSM was captured by the first 18 PCs, only 5 PCs were used in the reconstruction algorithm. This selection balanced reconstruction accuracy and computational efficiency. To validate this choice, the reconstruction algorithm was also tested with 10 and 15 PCs, but no relevant differences in reconstruction performance were observed, supporting the decision to retain 5 PCs. On average, reconstructing a scapula shape from skin landmarks using 5 PCs required approximately 12 min, with computation time increasing by a factor of 3.2 when using 15 PCs. Although the generalization and specificity errors were higher in magnitude compared to previous studies, the behavior of these metrics was consistent with the literature [16, 22, 25], reinforcing the validity of the developed SSM. The higher reconstruction errors likely stem from the lower number of points used to represent the 3D scapulae and differences in the distance metrics applied.

The 12 skin-to-bone regression models showed moderate to strong coefficients of determination, ranging from 0.70 to 0.98. In comparison, using a similar approach for the lower limbs, Nolte et al. [18] reported lower coefficients of determination (0.17 to 0.71), which underscores the effectiveness of the developed regression models, especially considering the substantial soft tissue coverage of the scapula [28]. Of the 17 initially defined predictors, a maximum of 5 predictors was included in each model. Overall, even though the data used were not scaled, most coefficients ranged between 0 and 1, except for the intercept values, which were modified according to the coefficients chosen for the regressors. The regression equations for the *x*, *y*, and *z* coordinates of the AA bone landmark, the *x* and *y* coordinates of the TS bone landmark, and the *x* coordinate of the AI bone landmark do not directly depend on their corresponding skin coordinates, as these were excluded from the models for being null in the defined scapula body-fixed reference frame. However, these landmarks were still represented indirectly through other predictors, such as inter-landmark distances. The *x* and *y* regression equations for the AA bone landmark were the only ones to identify sex as a significant predictor, suggesting a relationship between acromion shape and sex-related physiological differences. This is consistent with findings from Soltanmohammadi et al. [25], who reported significant differences in the posterior tilting of the acromion between male and female scapulae using a SSM.

The application of the skin-to-bone regression models to both training and testing datasets yielded prediction error distributions with a maximum median error of 4 mm. In the test set, all models—except those predicting the *x* and *z* coordinates of the AI landmark and the *y* and *z* coordinates of the AC landmark—achieved maximum errors of approximately 4 mm or less. The higher prediction errors observed for the AC models likely stem from the greater anatomical variability of this landmark [16, 25]. For the AI models, the reduced performance may be due to the greater inter-subject variability in soft tissue thickness between the bone and skin representations of the AI landmark, compared to the other landmarks. As most models presented outliers, an outlier analysis was performed, where each subject was evaluated regarding sex, age, weight, and height, as well as overall position of bone and skin landmarks. This analysis revealed that most outliers were associated with subjects with high BMIs, mainly due to their high weight, ranging between 95 and 120 kg. Additionally, two outliers were associated with low-BMI subjects, mainly due to their low weight. The identified subjects also represented outliers for the skin landmark variables, reflecting how variations in body weight can significantly alter the amount of soft tissue between bone and skin landmarks. To better account for the influence of extreme BMIs on landmark prediction, future improvements could include segmenting the dataset into BMI categories and developing separate models for each group, or applying more robust regression techniques.

While moderate to strong coefficients of determination were obtained across regression models, prediction errors were not equally distributed. The AI landmark, for example, exhibited larger errors than the AA and AC landmarks despite comparable coefficients of determination. This discrepancy is likely related to the AI's deeper anatomical location within the body, which may result in greater soft tissue variability and thus reduced prediction accuracy. These results reflect the fact that coefficients of determination only indicate the proportion of variance explained by the regression models but not the absolute magnitude of prediction errors. Both model fit and absolute prediction accuracy should be considered when evaluating regression performance.

To evaluate the performance of the reconstruction algorithm, scapula shapes were reconstructed using both digitized bone landmarks and bone landmarks predicted by the skin-to-bone regression models. The L2L errors for individual bone landmarks revealed higher interquartile ranges and whiskers for the AA and AC landmarks, likely due to the larger biological variability of the acromion compared to other regions of the scapula [16]. Even though median L2L errors for the shapes reconstructed from digitized and predicted bone landmarks were within the same range, those derived from the predicted bone landmarks presented more outliers. This increase in outliers can likely be attributed to errors introduced by the skin-to-bone regression models, which increased variability.

The S2S reconstruction errors, calculated across all 6000 points of the scapula shapes, were consistently low regardless of whether digitized or predicted bone landmarks were used. Notably, the Kruskal–Wallis test revealed no significant differences between the reconstruction errors of these two approaches, demonstrating the robustness of the reconstruction algorithm based on skin landmarks. For comparison, Mutsvangwa et al. [17] reconstructed scapula shapes by fitting SSM-generated scapula shapes to 3D coordinates of the AI, AA, and PC bone landmarks extracted from 2D X-ray images, reporting average S2S errors of 3.20 mm for the training dataset and 4.28 mm for the testing dataset. Their larger S2S errors may result from using only three landmarks, lacking information about scapular deformation in regions near the missing landmark. Similarly, Nolte et al. [18] reported median S2S errors of 2.60 mm and 2.90 mm for reconstructing femur and tibia/fibula shapes, respectively, from skin landmarks, comparable to the results obtained in this study.

While this study advances the computational modeling of the scapula, several limitations should be acknowledged. The sample size was small, with only 56 samples included. Increasing the sample size in future studies could enhance robustness of the skin-to-bone regression models and the SSM, potentially improving generality and specificity metrics. Nonetheless, the sample size considered in this study is comparable to that of other studies [16, 18, 21, 22]. The SSM developed in this study used a random shape from the training dataset as the reference shape. To minimize potential biases in the reconstruction process, future studies should consider using an average or consensus shape derived from all available training shapes as the reference [16]. Additionally, the bone and skin landmarks used in this study were idealized, as they were digitized from DICOM imaging data, and the algorithm’s accuracy was not tested using noisy motion capture data. Moreover, the skin-to-bone regression models were based on landmarks measured in a supine position, whereas motion capture laboratories typically collect landmarks in an upright position. This positional discrepancy likely affects the soft tissue offsets between skin and bone landmarks, which should be accounted for in future research. Finally, the coordinates of each bone landmark were predicted independently using univariate regression models, which simplifies computation but may neglect anatomical correlations between the three spatial axes.

In conclusion, this study demonstrated that scapula shape reconstructions based on a limited set of landmarks, often obtainable through motion capture systems, can effectively generate subject-specific computational models without relying on additional medical imaging. This approach may serve as a practical and efficient alternative to traditional methods of biomechanical modeling, with potential applications in both clinical and research contexts.

## Supplementary Information

Below is the link to the electronic supplementary material.

Supplementary file (DOCX 686 kb)
