# Supplementary material for: Reconstruction of Scapula Bone Shapes from Digitized Skin Landmarks Using Statistical Shape Modeling and Multiple Linear Regression
Source: Ann Biomed Eng. 2025 Jun 19;53(9):2239–50. doi: 10.1007/s10439-025-03768-1 (PMC12391217; doi:10.1007/s10439-025-03768-1)
Supplement: Supplementary file 1 — Supplementary file (DOCX 686 kb) [file 10439_2025_3768_MOESM1_ESM.docx]

**Reconstruction of scapula bone shapes from digitized skin landmarks using statistical shape modeling and multiple linear regression**

Augusto Marques, João Folgado, Carlos Quental

IDMEC, Instituto Superior Técnico, Universidade de Lisboa

**Supplementary material**

**1. Materials and methods**

**1.1 Pre-Processing**

Fifty-six scapula models, resulting from the image segmentation process, were decimated and smoothed using a reduction factor of 90% and a Laplacian filter with 1000 iterations, respectively. The smoothed meshes were reorganized to have isotropic vertices, and further decimated until the number of vertices equaled the vertex count selected. The vertex count for the scapula was set to *N_p_* = 6000. After this, a point cloud was generated from each mesh vertices, therefore defining a shape, **x**, as one vector made of *N_p_* points, i.e:

| $\mathbf{x}=\left( x_{1},y_{1},z_{1},\ldots,x_{N_{p}},y_{N_{p}},z_{N_{p}} \right)^{T}$. | (1) |
| --- | --- |

Centroid size scaling was performed to facilitate the correspondence between shapes.^6^ This was done by computing the square root of the sum of the squared distances between each point and the centroid. All shapes were initially aligned using their principal axes of inertia.

**1.2 Registration Procedure**

The Iterative Closest Point (ICP) algorithm was used for rigid registration of scapula shapes to the reference shape, where rotation and translation transformations were applied to approximate each subject shape to the reference as best as possible. Next a non-rigid registration was conducted using the Coherence Point Drift (CPD) algorithm.^7^ This step output the point-to-point correspondence between each subject shape and the reference shape, thus allowing the computation of dissimilarity metrics between the two. A random shape was chosen as reference. To implement the ICP and CPD algorithms, the Open3d and PyCPD libraries were used, respectively.^3,11^

**1.3 Principal Component Analysis**

The displacements for each pair of corresponding points, between each subject shape and the reference shape, were computed such that:

| $\Delta\mathbf{x}_{i}=\mathbf{x}_{i}-\mathbf{x}_{ref}$, $\forall i=1,\ldots,N_{s},$ | (2) |
| --- | --- |

where *N_s_* = 39, in accordance with the 70%-30% ratio considered for the training and test shape datasets. A displacement matrix was computed with columns being the coordinates x, y and z of all considered points, and lines being the coordinates’ displacement, for a given shape out of the 39. The displacement matrix was given as input to the PCA algorithm, allowing the computation of the corresponding covariance matrix. The covariance matrix **S** represents how a given point tends to vary across the population of scapula shapes, and how much this same variation tends to affect the variation of all other points considered. This matrix is mathematically computed as follows:

| $\mathbf{S}=\frac{1}{N_{s}-1}\sum_{i=1}^{N_{s}} \left( \Delta\mathbf{x}_{i}-\bar{\Delta\mathbf{x}} \right)^{T}\left( \Delta\mathbf{x}_{i}-\bar{\Delta\mathbf{x}} \right)$, | (3) |
| --- | --- |

where the average point displacements $\bar{\Delta\mathbf{x}}$ are given by:

| $\bar{\Delta\mathbf{x}}=\frac{1}{N_{s}}\sum_{i=1}^{N_{s}} \Delta\mathbf{x}_{i}$. | (4) |
| --- | --- |

Performing an eigen decomposition on covariance matrix **S** outputs the eigenvectors, $\boldsymbol{\alpha}$, and eigenvalues, $\boldsymbol{\lambda}$. Each eigenvector represents a different way the scapula shape can vary with statistical significance, with an associated variation, $\lambda$, resulting in a set of principal components often called modes of variation, *m*. Assuming a normal distribution in the variation of scapula shapes, the eigenvalues can be used to obtain the standard deviation associated with a given mode of variation. By applying limits of $\pm3\sqrt{\lambda_{m}}$, any computed shape is ensured to belong to the population of ground truth shapes with a confidence interval of 99.7%. A new shape, **x**_new_, is approximated by a linear combination of the first *M* modes of variation, like so:

| $\mathbf{x}_{new}=\mathbf{x}_{ref}+\bar{\Delta\mathbf{x}}+\sum_{m=1}^{M} \kappa_{m}\sqrt{\lambda_{m}}\boldsymbol{\alpha}_{m}$, | (5) |
| --- | --- |

where the mean shape of the SSM is given by the reference shape $\mathbf{x}_{ref}$ plus the average displacements $\bar{\Delta\mathbf{x}}$.^6^ In further sections, the focus is turned to the parameters $\kappa_{m}$, which mediate the amount of variation introduced by each of the *M* modes of variation into the mean shape. The vector of *M* normally distributed values, between −3 and +3, is henceforth defined as $\boldsymbol{\kappa}=\left( \kappa_{1},\kappa_{2},\ldots,\kappa_{M} \right)$.

**1.4 Validation metrics**

To validate the SSM created, standard performance metrics were considered, namely generalization, specificity and compactness.^4^

A compact shape model is a model that can accurately reconstruct new shapes with as little shape parameters as possible. Thus, compactness of the *M*-th principal component is defined as the cumulative explained variance, obtained by the covariance matrix decomposition, such that:^1^

| $C\left( M \right)=\frac{\sum_{m=1}^{M} \lambda_{m}}{\sum_{m=1}^{N_{s}} \lambda_{m}}\times100$. | (6) |
| --- | --- |

Specificity measures how good a given set of randomly generated shapes compares to the ground truth shapes. The specificity error, averaged over the total number of comparisons made, *N_C_*, between a random shape **x**′, constructed considering *M* principal components, and its closest ground truth shape **x**, both formed by *N_p_* points, is given by:^1^

| $S\left( M \right)=\frac{1}{N_{C}}\sum_{i=1}^{N_{C}} \sqrt{\frac{1}{N_{P}}\left\Vert\mathbf{x}_{i}^{,}\left( M \right)-\mathbf{x}_{i} \right\Vert^{2}}$. | (7) |
| --- | --- |

For each comparison, the random shape was compared to all the ground truth shapes, such that the closest to the random shape was that with the lowest error. In this study, a total of *N_C_* = 1000 random shapes and *N_p_* = 6000 points were used.

Finally, the generalization metric measures the ability of the constructed SSM to represent new shapes which are not part of the original training set. This is done by performing leave-one-out (LOO) tests on the training shapes. The reconstructed shape is computed using the solution of an optimization problem. The optimal amount of scapular variability $\boldsymbol{\kappa}^{*}$ is sought by minimizing the distance between the reconstructed and target shapes. The optimization problem was solved through the application of a genetic algorithm (GA) followed by a Truncated-Newton Constrained (TNC) algorithm, implemented via the pygad and scipy Python modules, respectively.^2,10^ Running the algorithms for *N_s_* shapes, i.e. performing *N_s_* LOO tests, the generalization metric was computed as the average reconstruction RMSE, along the *N_s_* shapes of the training dataset, between the excluded shape, **x**, and the best reconstructed shape computed using *M* principal components, $\mathbf{x}^{*}(M)$. The generalization metric is therefore given by:^1^

| $G\left( M \right)=\frac{1}{N_{s}}\sum_{i=1}^{N_{s}} \sqrt{\frac{1}{N_{p}}\left\Vert\mathbf{x}_{i}-\mathbf{x}_{i}^{*}(M) \right\Vert^{2}}$. | (8) |
| --- | --- |

**2. RESults and discussion**

**2.1 Validation metrics**

The results regarding the validation metrics of the SSM are depicted in Figure S1.

| 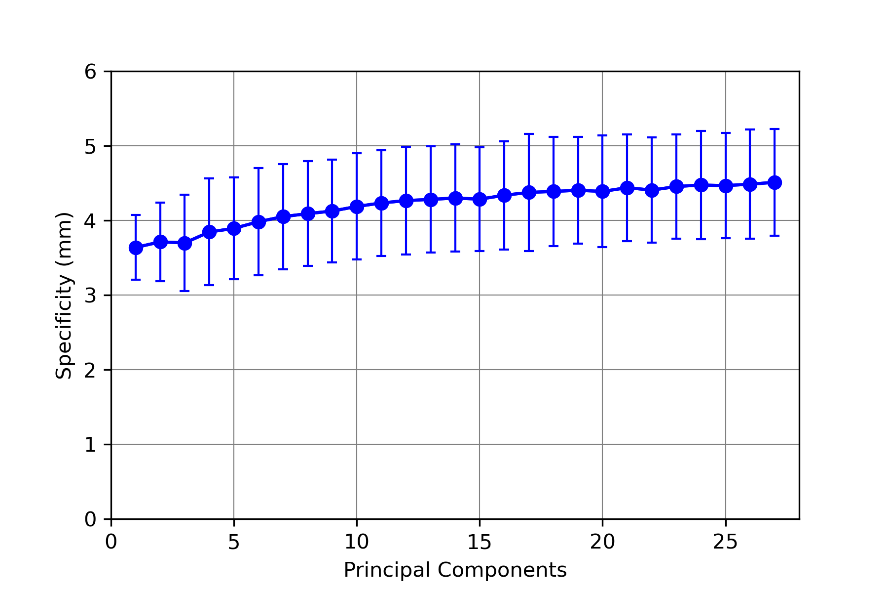 |
| --- |
| (a) |
| 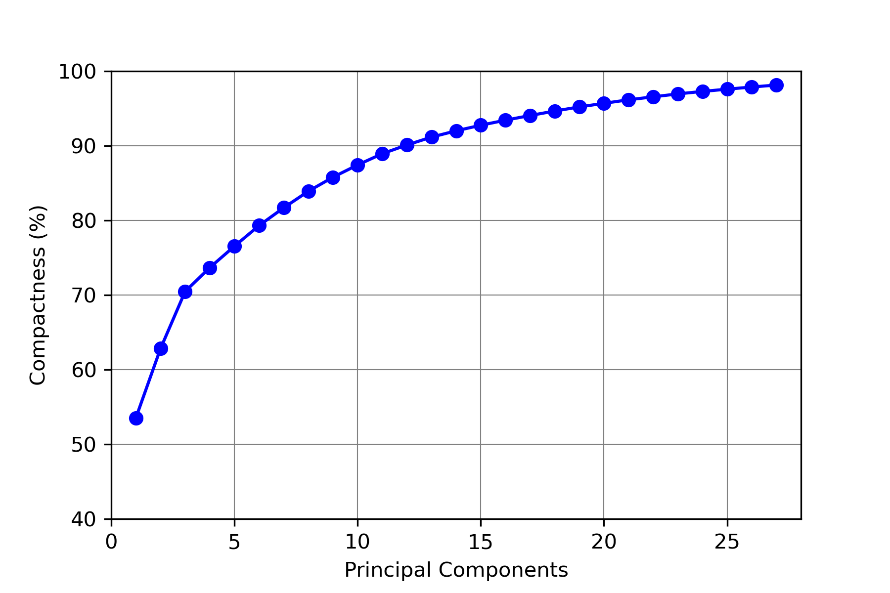 |
| (b) |
| 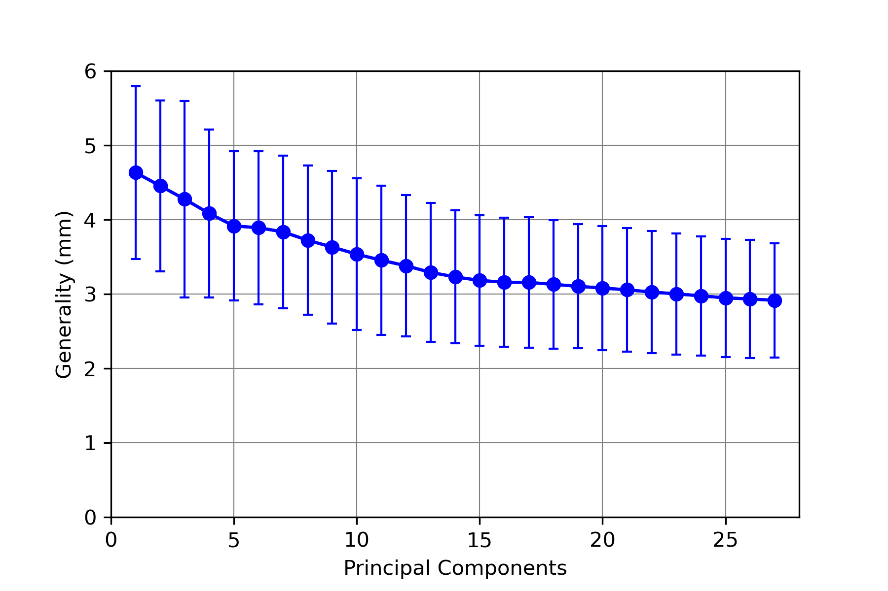 |
| (c) |

**Figure S1**. Validation metrics of (a) compactness, (b) generality and (c) specificity. The three metrics are computed for 27 principal components, representing a maximum of 98% cumulative variance.

The cumulative variance increases with the number of principal components. Compactness evolution through the number of PCs is slower in comparison with the literature seen for the scapula, with Mutsvangwa et al.,^6^ Soltanmohammadi et al.,^9^ and Salhi et al.^8^ describing 90%, 91% and 95% scapula variability using 7, 9 and 15 PCs, respectively. A different dataset of ground truth shapes might generate different PCs and consequently small deviations in the cumulative variance covered.

For generalization, scapula reconstruction errors have a decreasing evolution. Maximum and minimum RMSEs of 4.63 mm and 2.91 mm were identified, respectively. These values are higher in comparison to other studies. Mutsvangwa et al.^6^ achieved maximum and minimum RMSEs of 1.90 mm and 1.00 mm, whereas Salhi et al.^8^ reached 1.64 mm and 1.07 mm. This difference might be explained by the vertex count considered. Mutsvangwa et al.^6^ and Salhi et al.^8^ used 15000 points compared to the 6000 considered in this study. The higher generality errors observed in this study may have been influenced by the lower point cloud density, which likely led to increased distances between corresponding points. Furthermore, to reduce computational complexity, the same correspondence was considered between each reconstructed shape and the target shape, potentially amplifying point-to-point disparities.

Specificity presented a local minimum for the first PC, and increased intermittently along the number of PCs. The specificity values obtained range between 3.64 and 4.51 mm, approximately. Mutsvangwa et al.^6^ achieved specificity values between 1.40 mm and 1.60 mm, whereas Salhi et al.^8^ obtained values between 1.22 mm to 1.74 mm. The increase regarding this study could be due to the different vertex count, similarly to the generalization metric, and the fact that the correspondence between each random reconstruction and the target shape was considered as the correspondence between the mean shape and the target shape, for the sake of computational feasibility.

The number of PCs to retain is normally chosen according to those that cover a predefined percentage of cumulative variance,^4^ although such rule has been shown to be highly dependent on the sample size.^5^ According to Mei et al.^5^, for a fixed threshold of cumulative variance, there is an increase in the cut-off number of adequate PCs with an increased sample size, leading to the consideration of noise inducing PCs which do not cover important features of anatomical variation. Therefore the number of PCs was chosen according to the "knee" of the generality curve, where the curve flattens, representing the best trade-off between average reconstruction error and computational expense. Five PCs were initially considered to show the SSM scapular variation since it was the number of PCs chosen for the reconstruction algorithm, based on Figure S1b. Results acquired with 10 and 15 PCs showed similar results, thus five PCs were maintained.

**2.2 Scapula Reconstructions from Landmarks – Best and Worst**

Figure S2 shows the superimposition between the reconstructed and ground truth shapes for the best and worst reconstructions. The vertex count of 6000 points shows a good discretization of the scapula surface. Both reconstructed shapes show uniform and homogeneous forms, with the overall scapula shape being preserved. For the best case, accurate reconstructions were obtained for the subscapular and infraspinous fossae, the acromion, the coracoid process, the glenoid cavity, and the lateral border. Higher point-to-point distances are prominent along the superior border and between the superior angle and the root of the spine. For the worst case, only the subscapular and infraspinous fossae were accurately reconstructed. The remaining regions present elevated point-to-point distances, particularly for the coracoid process, the glenoid cavity, and the inferior part of the medial border.

| Anterior | Lateral | Posterior | Medial |
| --- | --- | --- | --- |
| 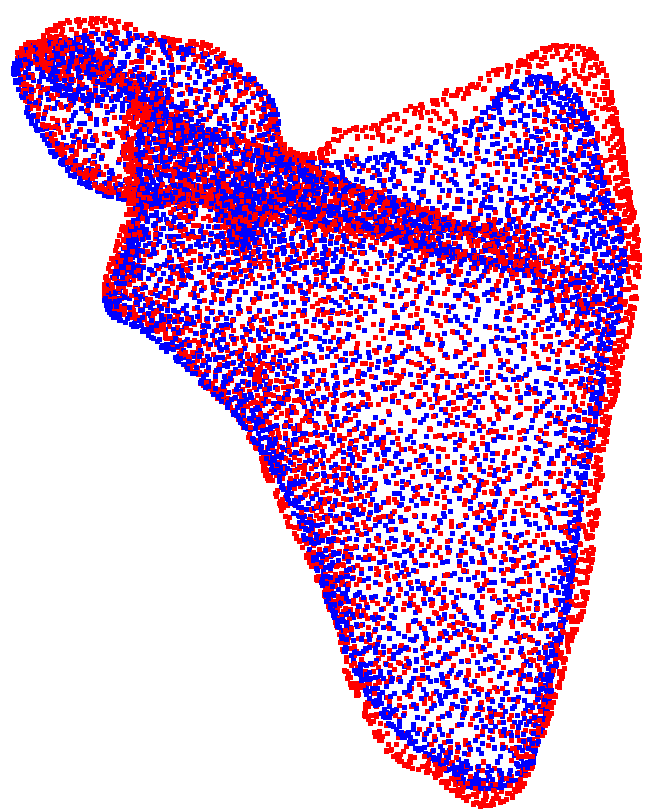 | 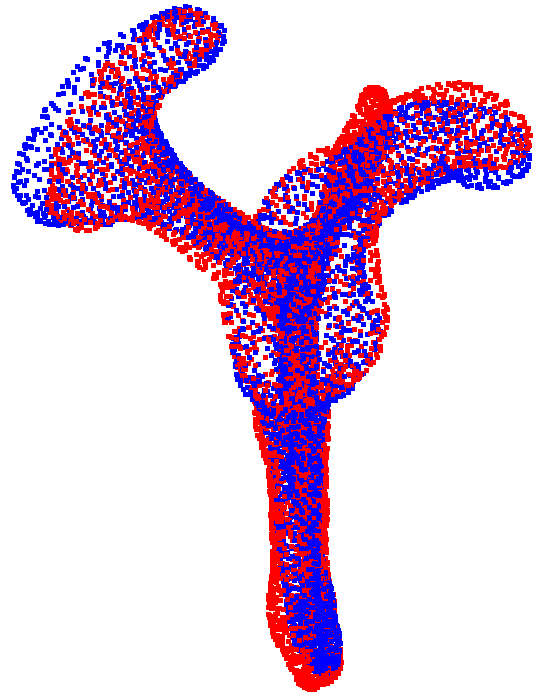 | 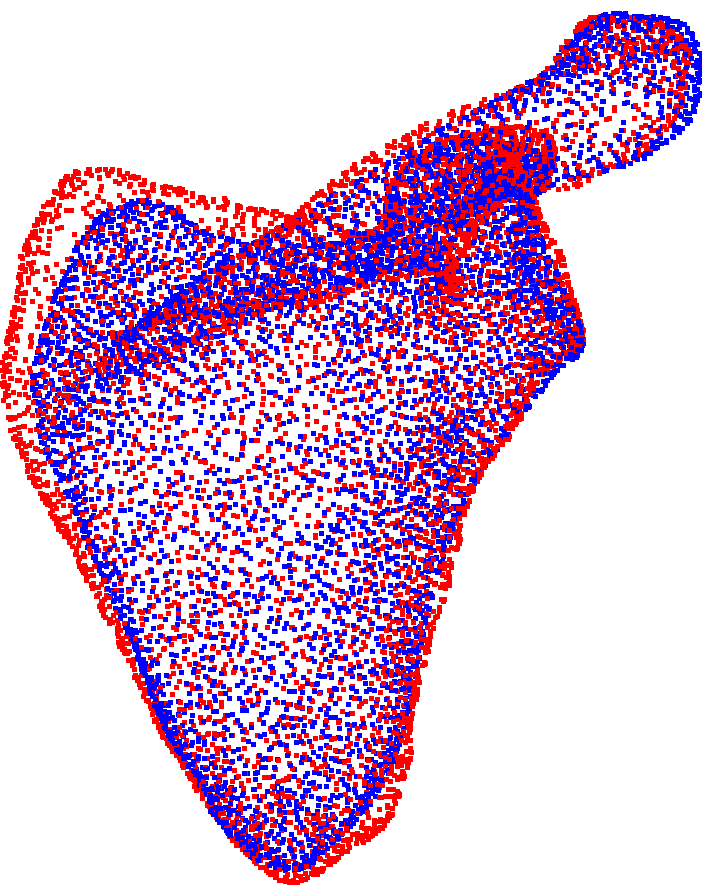 | 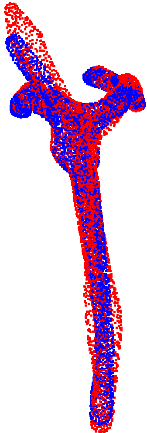 |
| 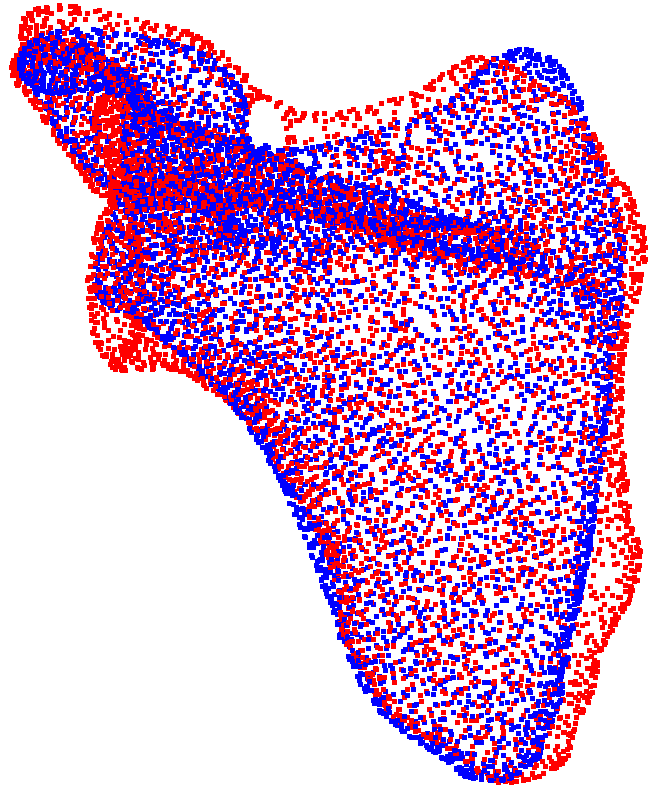 | 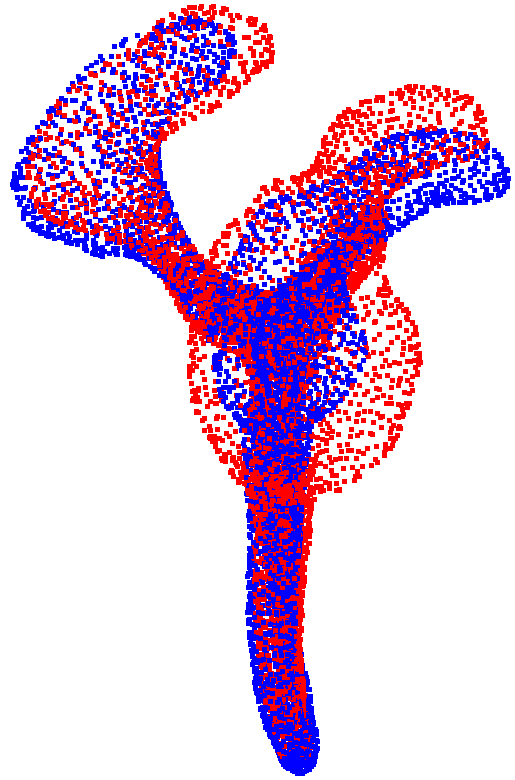 | 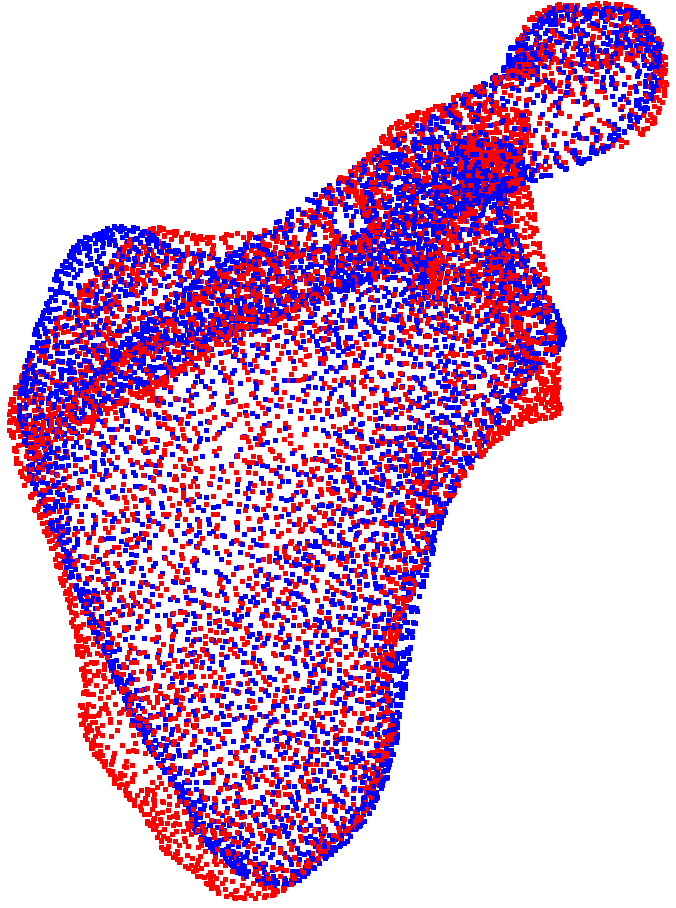 | 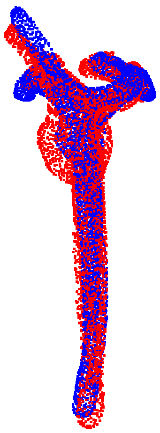 |

**Figure S2**. Best (top) and worst (bottom) scapula reconstruction (blue) superimposed with the respective ground truth shape (red), obtained with five principal components and from predicted bone landmarks, in the form of point clouds. Superimposed shapes are shown after rigid and non-rigid registration, thus representing the best alignment between the two shapes. Meshes are not shown since the superimposition prevents a good visualization of the shapes.

**References**

1. Audenaert, E. A., C. Pattyn, G. Steenackers, J. De Roeck, D. Vandermeulen, and P. Claes. Statistical Shape Modeling of Skeletal Anatomy for Sex Discrimination: Their Training Size, Sexual Dimorphism, and Asymmetry. *Front Bioeng Biotechnol* 7:, 2019.

2. Gad, A. F. PyGAD: an intuitive genetic algorithm Python library. *Multimed Tools Appl* 83:58029–58042, 2024.

3. Gatti, A. A., and S. Khallaghi. PyCPD: Pure NumPy Implementation of the Coherent Point Drift Algorithm. *J Open Source Softw* 7:4681, 2022.

4. Heimann, T., and H. P. Meinzer. Statistical shape models for 3D medical image segmentation: A review. *Med Image Anal* 13:543–563, 2009.

5. Mei, L., M. Figl, D. Rueckert, A. Darzi, and P. Edwards. Statistical shape modelling: How many modes should be retained? *2008 IEEE Computer Society Conference on Computer Vision and Pattern Recognition Workshops, CVPR Workshops* , 2008.doi:10.1109/CVPRW.2008.4562996

6. Mutsvangwa, T., V. Burdin, C. Schwartz, and C. Roux. An Automated Statistical Shape Model Developmental Pipeline: Application to the Human Scapula and Humerus. *IEEE Trans Biomed Eng* 62:1098–1107, 2015.

7. Myronenko, A., and X. Song. Point set registration: Coherent point drifts. *IEEE Trans Pattern Anal Mach Intell* 32:2262–2275, 2010.

8. Salhi, A., V. Burdin, A. Boutillon, S. Brochard, T. Mutsvangwa, and B. Borotikar. Statistical Shape Modeling Approach to Predict Missing Scapular Bone. *Ann Biomed Eng* 48:367–379, 2020.

9. Soltanmohammadi, P., J. Elwell, V. Veeraraghavan, G. S. Athwal, and R. Willing. Investigating the Effects of Demographics on Shoulder Morphology and Density Using Statistical Shape and Density Modeling. *J Biomech Eng* 142:, 2020.

10. Virtanen, P. *et al.* SciPy 1.0: fundamental algorithms for scientific computing in Python. *Nat Methods* 17:261, 2020.

11. Zhou, Q.-Y., J. Park, and V. Koltun. Open3D: A Modern Library for 3D Data Processing. , 2018.at <https://arxiv.org/abs/1801.09847v1>
